# Reconstruction of Acute Patellar Tendon Rupture after Patellectomy

**DOI:** 10.1155/2018/7549476

**Published:** 2018-04-01

**Authors:** Kenjiro Fujimura, Koji Sakuraba, Satoshi Kamura, Kiyoshi Miyazaki, Nobuo Kobara, Kazumasa Terada, Hisaaki Miyahara

**Affiliations:** ^1^Clinical Research Institute, National Hospital Organization, Kyushu Medical Center, Fukuoka, Japan; ^2^Department of Orthopaedic Surgery, National Hospital Organization, Kyushu Medical Center, Fukuoka, Japan

## Abstract

Acute rupture of the knee extensor mechanism after patellectomy is extremely rare. We present the case of a patient with acute patellar tendon rupture who had undergone patellectomy 53 years before. Twelve days after the injury, the ruptured patellar tendon was repaired with end-to-end suture. Postoperatively, we splinted the knee for 6 weeks but permitted the patient to walk without limiting weight bearing at 1 week postoperatively. At one-year follow-up, the patient is able to move his knee almost full range of motion and the Lysholm knee score is 81. The patient is satisfied with the outcome. This is the first report to treat acute rupture of the patellar tendon in a patient who had undergone patellectomy. Although careful rehabilitation is required, end-to-end suture might be an adequate surgical procedure for acute rupture of the knee extensor mechanism after patellectomy.

## 1. Introduction

The most frequent cause of failure of the knee extensor mechanism is patellar fracture, while ruptures of the patellar tendon or quadriceps tendon are comparatively rare. Patellar tendon ruptures are reportedly the least frequent cause of knee extension failure [1–4]. Moreover, as patellectomy is only performed when there are no other methods for reconstructing the patella [3, 5], this procedure is currently rarely performed. Thus, acute rupture of the knee extensor mechanism after patellectomy is extremely rare. In fact, there is only one case report on acute quadriceps tendon rupture after patellectomy [6], while acute patellar tendon rupture after patellectomy has not yet been reported in the English literature.

The present report describes the case of a 73-year-old male patient who ruptured his left patellar tendon 53 years following patellectomy. The treatment modality and the outcome are presented.

## 2. Case Report

A 73-year-old male had a traffic accident while riding his bicycle and hit his left knee on the ground. He presented at our hospital 4 days after the accident. He could walk without crutches but could not extend his knee against gravity. We palpated a subcutaneous depression in the left knee.

The patient was 165.5 cm tall, weighed 63.8 kg, and had a BMI of 23.3 kg/m^2^. He had previously experienced a comminuted left patellar fracture and underwent a total patellectomy when he was 20 years old. After the patellectomy, he had no complaints and had a full range of movement in the left knee. The preinjury Lysholm knee score was 90 [7]. At 57 years of age, the patient had received mitral valve replacement for regurgitation at another hospital and had been on anticoagulant therapy since then.

**Figure 1:**
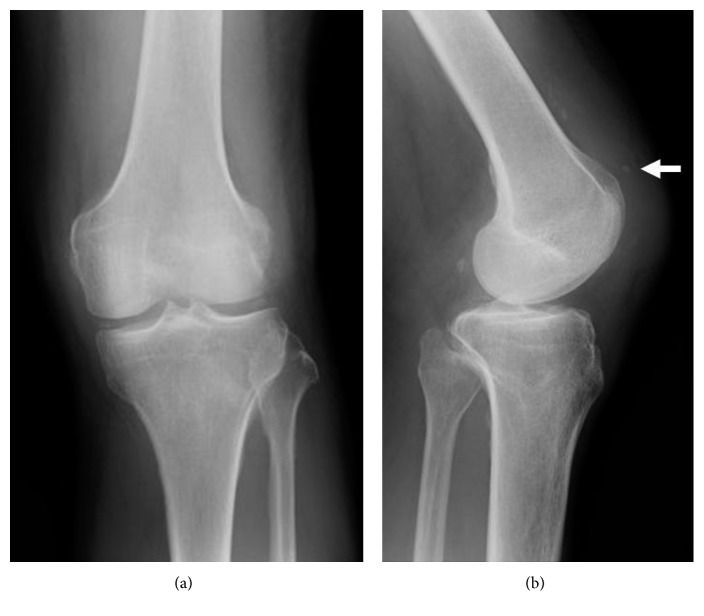
Plain radiographic anteroposterior view (a) and lateral view (b) of the left knee showing the absence of a patella and the presence of a small heterotopic calcification (white arrow) at the distal side of the quadriceps tendon.

**Figure 2:**
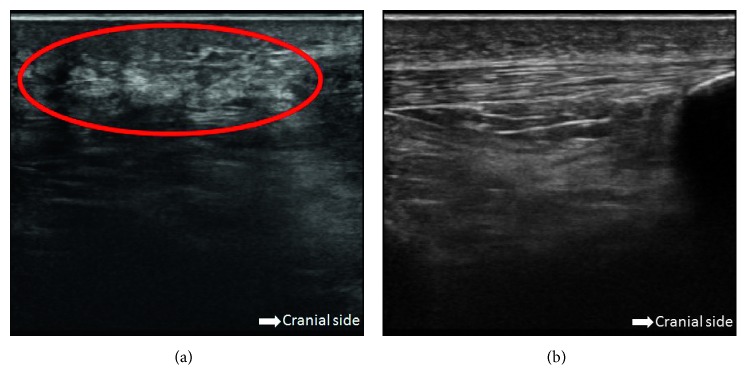
Ultrasound examination showed a loose patellar tendon (within red circle) (a) compared with the contralateral side (b). The right is the cranial side, and the patella can be seen on the far right in (b).

**Figure 3:**
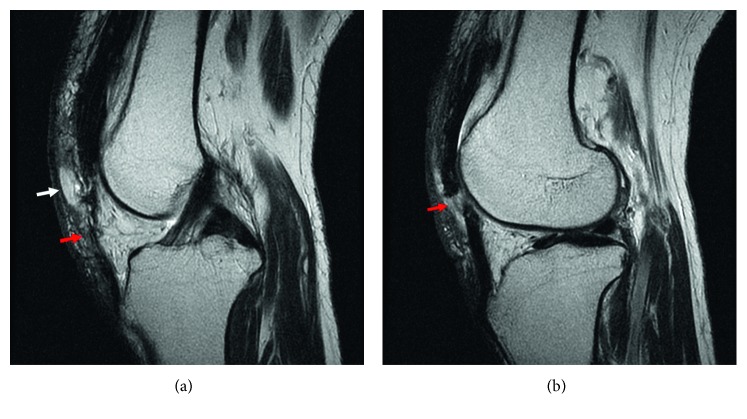
T2-weighted magnetic resonance imaging showed a loose patellar tendon (red arrow) and subcutaneous edema (white arrow) (a) and tendon disruption (red arrow) (b) at the proximal side of the patellar tendon.

Plain radiographic examination did not show any fracture of the left knee but detected the absence of the patella and a small heterotopic calcification at the distal side of the quadriceps tendon (Figure 1). Ultrasound examination showed a loose left patellar tendon compared with the contralateral side (Figure 2), although it could not identify the rupture site. Magnetic resonance imaging (MRI) revealed subcutaneous edema and tendon disruption at the proximal side of the left patellar tendon, which indicated patellar tendon rupture (Figure 3). We immediately immobilized the left knee with a splint and changed his anticoagulant therapy from warfarin to intravenous heparin.

**Figure 4:**
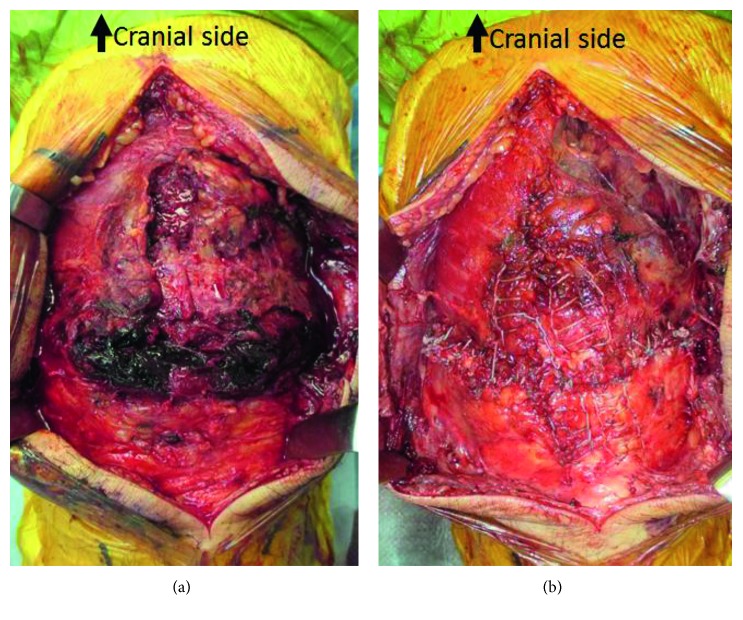
Intraoperative photographs. The top is cranial and the bottom is caudal. The patellar tendon and patellar retinaculum were completely ruptured. Hematoma filled the rupture site (a). We performed end-to-end suture with two Krackow locking stitches and figure-of-eight sutures (b).

Surgery to reconstruct the ruptured left patellar tendon was performed 12 days after the accident. We made a midline incision instead of an oblique incision along the previous scar and found a complete patellar tendon rupture with both medial and lateral patellar retinaculum rupture with about a 2.0 cm gap filled with a hematoma (Figure 4(a)). These ruptures were at the proximal side of the patellar tendon. We first washed and removed the hematoma and refreshed the ruptured tendon edge with scissors. The length of remained patellar tendon was about 5 cm. We then performed end-to-end suturing with two Krackow locking stitches with #2 Hifi high-strength suture (CONMED, NY, USA) and added approximately twenty figure-of-eight sutures with #0 Hifi high-strength suture (Figure 4(b)).

Postoperatively, the left knee was protected with a splint for 6 weeks. The patient was permitted to walk without limiting weight bearing at 1 week postoperatively. After 6 weeks, knee flexion exercise was started, but the knee was protected in extension with a knee brace during walking for another 6 weeks. At postoperative 3 months, the patient could walk without any difficulty and could almost fully flex his left knee but had an extensor lag of 20° and left quadriceps muscle atrophy. Currently (at 1 year postoperatively), the patient can extend his knee with almost no extension lag and can flex fully but has persistent quadriceps muscle atrophy. The Lysholm knee score at 1 year postoperatively is 81. The patient is satisfied with the outcome.

Written consent was obtained from the patient for publication of the study.

## 3. Discussion

The knee extensor mechanism is comprised of the patella, quadriceps tendon, and patellar tendon. Loss of the knee extensor mechanism is most commonly caused by patellar fracture. Patellar tendon rupture is the rarest cause of knee extensor failure, and this mainly occurs due to indirect trauma in patients under 40 years of age [1–4]. Ruptures usually occur in weakened tendons that have degenerative changes caused by iterative microtrauma, local corticosteroid injections, or systemic diseases such as diabetes, thyroid disorders, renal disease, hyperlipidemia, and systemic inflammatory diseases [1–3]. Traumatic patellar tendon rupture is also reported [2], as seen in the present case. Currently, total patellectomy is considered the final method for treating severe osteomyelitis, severe comminuted fractures, or open fracture with bone loss, as clinical outcomes are unsatisfactory [3, 5]. Hence, it is extremely rare to encounter rupture of the knee extensor mechanism after patellectomy, and only few cases have been reported [6, 8–10]. Shanmugam and Maffulli reported the first case of acute quadriceps tendon rupture in a patient with patellectomy [6]. There are three other reports on rupture of the patellar tendon after patellectomy [8–10]; all of these ruptures occurred at the site of the tibial tubercle and were successfully treated at the chronic phase with either an Achilles tendon allograft [8], the iliotibial band from the contralateral side [9], or the gracilis and semitendinosus tendons [10]. This is the first report of treatment of an acute rupture of the patellar tendon in a patient who had previously undergone patellectomy. The present patient incurred this rupture by direct trauma, similarly to the patient in the report by Shanmugam and Maffulli [6].

Ultrasound and MRI examinations are reportedly better at diagnosing chronic patellar tendon ruptures compared with acute cases [3]. In the present case, ultrasound examination could not reveal the rupture site but detected only the loose patellar tendon; however, MRI examination was useful to detect the existence of a rupture at the patellar tendon side. Although loss of the knee extensor mechanism can be diagnosed relatively easily by palpation of a subcutaneous depression and failure of active knee extension [3], it might be difficult to determine the location of the rupture site in patients without a patella. The present case findings suggest that imaging examinations are also effective to clarify the details of the extensor mechanism of the knee.

Surgical treatment is required for patellar tendon rupture. In particular, end-to-end suture is selected for full-body rupture. Although reinforcement frames are often added to support the suture and avoid rerupture [2, 3], we selected end-to-end suture without reinforcement frames in the present case, as Shanmugam and Maffulli reported the successful use of this method [6]. They performed continuous locked suture with heavy absorbable sutures [6]; however, we selected two Krackow locking stitches with approximately twenty figure-of-eight sutures with nonabsorbable suture material, as we considered that this would provide adequate strength. Shanmugam and Maffulli examined the teared tendon margins histopathologically and found chronic hypoxic tendon degeneration [6]. Although we did not perform such an analysis, we considered that the present case would likely have had the same findings. Hence, we also resected and refreshed the tendon margin to prevent failure of the suture site.

In terms of the timing of surgery, Siwek and Rao reported that the result of delayed repair (more than 2 weeks after injury) was worse than that of immediate repair [11]. Another report recommended that the repair should be performed within 1 week of injury to achieve satisfactory results, and they performed surgery within 24 hours of the injury [6]. However, there are cases in which surgery cannot be performed immediately because of comorbidities or anticoagulant therapy, such as in the present case. Although some studies report that early mobilization results in a favorable outcome [12–14], strict immobilization with a walking cast is generally recommended to avoid suture failure. Previous reports have recommended a duration of immobilization of at least 1 month [3] and at least 6 weeks [2]. Moreover, Langenhan et al. found no significant differences between limited versus early functional rehabilitation protocol after surgical repair of quadriceps tendon rupture [15]. Our patient could have started knee flexion exercise earlier, but we immobilized his knee with a splint for 6 weeks because we selected elective surgery.

This is the first report of treatment of acute rupture of the patellar tendon in a patient who had undergone patellectomy, and the outcome was satisfactory. From our patient's favorable result and as reported previously [6], end-to-end suture without reinforcement frames is adequate for treating acute phase rupture of the extensor mechanism of the knee, even in patients who have undergone patellectomy.
